# Tasman-PCR: a genetic diagnostic assay for Tasmanian devil facial tumour diseases

**DOI:** 10.1098/rsos.180870

**Published:** 2018-10-03

**Authors:** Young Mi Kwon, Maximilian R. Stammnitz, Jinhong Wang, Kate Swift, Graeme W. Knowles, Ruth J. Pye, Alexandre Kreiss, Sarah Peck, Samantha Fox, David Pemberton, Menna E. Jones, Rodrigo Hamede, Elizabeth P. Murchison

**Affiliations:** 1 Transmissible Cancer Group, Department of Veterinary Medicine, University of Cambridge, Madingley Road, Cambridge CB3 0ES, UK; 2 Animal Health Laboratories, Mount Pleasant Laboratories, Tasmanian Department of Primary Industries, Parks, Water and the Environment, Prospect, Tasmania 7250, Australia; 3 Menzies Institute, University of Tasmania, 17 Liverpool Street, Hobart, Tasmania 7000, Australia; 4 Department of Primary Industries, Parks, Water and the Environment (DPIPWE), Save the Tasmanian Devil Program, Tasmania 7000, Australia; 5 Toledo Zoo, 2605 Broadway, Toledo, OH 43609, USA; 6 School of Natural Sciences, University of Tasmania, 55 Private Bag, Hobart, Tasmania 7000, Australia

**Keywords:** Tasmanian devil, transmissible cancer, diagnostic test, devil facial tumour disease

## Abstract

Tasmanian devils have spawned two transmissible cancer clones, known as devil facial tumour 1 (DFT1) and devil facial tumour 2 (DFT2). DFT1 and DFT2 are transmitted between animals by the transfer of allogeneic contagious cancer cells by biting, and both cause facial tumours. DFT1 and DFT2 tumours are grossly indistinguishable, but can be differentiated using histopathology, cytogenetics or genotyping of polymorphic markers. However, standard diagnostic methods require specialist skills and equipment and entail long processing times. Here, we describe Tasman-PCR: a simple polymerase chain reaction (PCR)-based diagnostic assay that identifies and distinguishes DFT1 and DFT2 by amplification of DNA spanning tumour-specific interchromosomal translocations. We demonstrate the high sensitivity and specificity of this assay by testing DNA from 546 tumours and 804 normal devils. A temporal–spatial screen confirmed the reported geographic ranges of DFT1 and DFT2 and did not provide evidence of additional DFT clones. DFT2 affects disproportionately more males than females, and devils can be co-infected with DFT1 and DFT2. Overall, we present a PCR-based assay that delivers rapid, accurate and high-throughput diagnosis of DFT1 and DFT2. This tool provides an additional resource for devil disease management and may assist with ongoing conservation efforts.

## 1. Introduction

Tasmanian devils (*Sarcophilus harrisii*) are marsupial carnivores endemic to the Australian island of Tasmania. This species is affected by two transmissible cancers, known as Tasmanian devil facial tumour 1 (DFT1) and Tasmanian devil facial tumour 2 (DFT2). DFT1 and DFT2 manifest as facial tumours collectively known as devil facial tumour disease (DFTD) and are transmitted between animals by the direct transfer of living allogeneic cancer cells, probably by biting [1,2]. Thus, DFT1 and DFT2 are two independent malignant clones, one of which arose from the somatic cells of an individual female devil (DFT1), the other from a male (DFT2), and which both now propagate through the devil population via transfer of contagious cancer cells [2–4]. DFT1 was initially reported in 1996 in northeast Tasmania and is now widespread across the island, causing significant declines in devil populations [5–7]. DFT2, on the other hand, which was first observed in 2014, has been reported in only five animals, all located in the Channel, an approximately 550 km^2^ peninsula in Tasmania's southeast [2].

DFTD tumours caused by DFT1 and DFT2 are grossly indistinguishable, but are histologically, cytogenetically and genetically distinct [2,8]. Differential diagnosis of DFT1 and DFT2 is routinely performed with histopathology and immunohistochemistry [2,9–11]. Diagnosis can also be confirmed using cytogenetics, as DFT1 and DFT2 have markedly different karyotypes [1,2]. Genetic methods, based on microsatellite and major histocompatibility complex (MHC) genotypes, have also been used to distinguish DFT1 and DFT2 [2]. However, the presence of host tissue within DFT1 and DFT2 tumour biopsies, as well as allelic variation across tumour and host populations, often leads to ambiguity with the interpretation of such genotypes.

DFT1 and DFT2 are foreign tissue grafts in their hosts and must escape the allogeneic immune system. DFT1 cells down-regulate MHC class I, perhaps contributing to this clone's low immunogenicity [12]; the mechanism of DFT2 immune avoidance is unknown. Interestingly, however, whereas DFT1 equally affects males and females [9], all five devils diagnosed with DFT2 have been male [2].

DFT1 was first detected in the Channel Peninsula in 2012, shortly before the discovery of DFT2 there in 2014 [2]. Thus, DFT1 and DFT2 have overlapping host ranges within the Channel Peninsula, and in the period from December 2012 to June 2015, seven cases of DFT1 and five of DFT2 were reported in the area [2]. While it is known that a devil can carry two strains, or subclones, of DFT1, derived from different infections [4], it remains unknown whether a host can possess both DFT1 and DFT2 tumours simultaneously.

Transmissible cancers have been rarely observed in nature. There are currently eight known naturally occurring transmissible cancers, five of which affect various species of bivalve molluscs [2,13–15]. In 1996, when DFT1 was first observed, only one other example was known, a sexually transmitted venereal tumour in dogs [15]. Furthermore, there was no evidence of devil tumours similar to DFTD prior to 1996 [5,9]. Thus, the discovery of DFT2 was surprising, and suggests that Tasmanian devils may be particularly vulnerable to the emergence of transmissible cancers [8]. There is a possibility that additional DFT clones may be present or may have previously existed in the devil population, but escaped detection. Moreover, the extent of the DFT2 range beyond the Channel Peninsula is not yet confirmed.

The identification of two transmissible cancers within an interval of 18 years raises important questions about Tasmanian devils and their susceptibility to transmissible cancers. Here, we describe the design and validation of a genetic diagnostic test for confirmation of DFT1 and DFT2 in devils. We use this ‘Tasman-PCR’ test to examine the range of DFT2 and to confirm the absence of additional DFT clones within a cohort of 546 tumours collected between 2003 and 2016 from more than 69 locations around Tasmania.

## 2. Results

### 2.1. Development of PCR-based markers for DFT1 and DFT2

**Figure 1.**
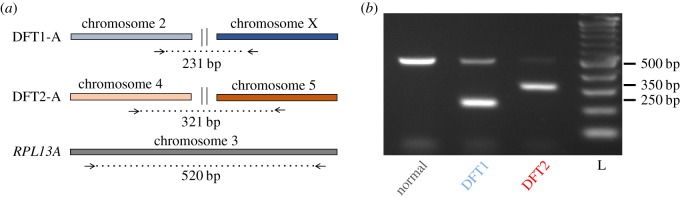
Tasman-PCR, a DFTD triplex diagnostic PCR. (*a*) Design of triplex PCR using primers spanning DFT1-A, DFT2-A and *RPL13A*. (*b*) Gel electrophoresis showing amplification products from normal devil DNA, as well as from DFT1 and DFT2 DNA. L, ladder.

DFT1 and DFT2 are both clonal lineages, hence somatic variants that were acquired early during each lineage's history may present useful markers for polymerase chain reaction (PCR)-based diagnostic assays. Interchromosomal translocations are particularly effective markers, as the presence or absence of an amplification product is the screening endpoint. We assessed a set of interchromosomal structural variants that had been identified and validated in two DFT1 and in two DFT2 genomes, respectively, but not found in 34 normal devil genomes [8]. Of these, we selected two putative DFT1-specific markers (DFT1-A and DFT1-B) and two putative DFT2-specific markers (DFT2-A and DFT2-B) for further screening (table 1). A triplex PCR assay, named Tasman-PCR, was then optimized, containing primers specific for DFT1-A (231 base pairs, bp), DFT2-A (321 bp), as well as positive control primers amplifying the endogenous *RPL13A* locus (520 bp) [12] (figure 1).

**Table 1.** Coordinates and breakpoint features for structural variants specific to DFT1 and DFT2 used in screen. Coordinates are relative to the Tasmanian devil reference genome Devil_refv7.0 [4]. [+] and [−] refer to strand.

clone	marker	coordinate 1	coordinate 2	breakpoint features
DFT1	DFT1-A	Chr2_GL841420 : 441799 [+]	ChrX_GL867598 : 2128339 [−]	blunt end
	DFT1-B	Chr1_GL834488 : 1096676 [−]	Chr4_GL856767: 219384 [−]	microhomology (AAAA)
DFT2	DFT2-A	Chr4_GL856969 : 2309705 [+]	Chr5_GL861630 : 40293 [+]	microhomology (AG)
	DFT2-B	Chr4_GL856736 : 2811567 [+]	Chr5_GL861700: 1309714 [+]	untemplated sequence insertion (ATAACTCATAATAGTACAT)

### 2.2. Normal devil screen

Interchromosomal translocations are more common in cancer than in germline DNA. However, it is possible that DFT1-A and/or DFT2-A were in fact inherited polymorphisms present in the constitutive genomes of the individual devils that spawned the respective clones, and hence may also be found in the genomes of some normal devils in the population. In order to assess this, we screened Tasman-PCR across normal DNA from 804 devils sampled between 2003 and 2016 from more than 65 locations in Tasmania, including both non-diseased individuals as well as devils infected with DFT1 and/or DFT2 (table 2; electronic supplementary material, table S1). DNA from three devils (0.4%) was faintly positive for DFT1-A, and no devils amplified DFT2-A (table 2; electronic supplementary material, table S1). We further assessed the three DFT1-A-positive DNA samples by amplifying a panel of microsatellite length polymorphisms. All three samples amplified three or more alleles at one or more loci, suggesting DNA contamination (electronic supplementary material, table S2A). Furthermore, all three DNA samples which were positive for DFT1-A also amplified DFT1-B (table 1), further suggesting the possibility that these normal samples may have been contaminated by DFT1 DNA. Altogether, these results suggest that the tumour DNA markers used in our triplex assay are highly specific for DFT1 and DFT2, and are likely to be somatic translocations that arose subsequent to neoplastic transformation in these two clones.

**Table 2.** Devils and tumours screened with Tasman-PCR. DFTD status was confirmed through histopathology, cytogenetics and/or microsatellite genotyping. DFT1/DFT2 status of DFTD suspected tumours was unknown. ‘Samples’ refers to individuals (normal devils) or to tumours (DFTD confirmed, DFTD suspected, non-DFTD tumour confirmed or suspected). *, samples showed signs of DFT1 tumour DNA contamination (see text). Further information about devils and tumours is available in electronic supplementary material, tables S1 and S3.

	tumours (individuals)	DFT1-A-positive samples	DFT2-A-positive samples	DFT1-A and DFT2-A-negative samples
normal devils	− (804)	3 (0.4%)*	0 (0%)	801 (99.6%)
DFTD confirmed	344 (259)	321 (93.3%)	10 (2.9%)	13 (3.8%)
DFT1 confirmed	334 (251)	321 (96.1%)	0 (0%)	13 (3.9%)
DFT2 confirmed	10 (8)	0 (0%)	10 (100%)	0 (0%)
DFTD suspected	175 (114)	158 (90.3%)	5 (2.9%)	12 (6.9%)
non-DFTD tumour confirmed or suspected	27 (25)	0 (0%)	0 (0%)	27 (100%)

### 2.3. Analysis of confirmed DFTD tumours

We used the triplex assay to screen 344 confirmed DFTD (DFT1 and DFT2) tumours (table 2; electronic supplementary material, table S3A). The tumours were collected between 2003 and 2016 from 56 locations in Tasmania, and DFTD status was confirmed through histopathology, cytogenetics and/or microsatellite genotyping [9,10,16]. In total, 331 of 344 (96.2%) confirmed DFTD tumours were positive for either DFT1-A or DFT2-A (321 DFT1-A positive, 10 DFT2-A positive), and no tumours were positive for both DFT1-A and DFT2-A (table 2; electronic supplementary material, table S3A).

We considered the following three explanations for the 13 remaining confirmed DFT1 tumours which did not amplify DFT1-A (table 2; electronic supplementary material, table S3A): (i) they belong to a DFT1 subclone which diverged from a clonal ancestor prior to DFT1-A being acquired; (ii) they belong to a DFT1 subclone which lost DFT1-A, or acquired mutations within the DFT1-A primer binding sites or (iii) the biopsy used for DNA extraction included only non-neoplastic host tissue, or included DFTD cells at levels that were undetectable under our PCR and gel electrophoresis conditions. To distinguish between these three possibilities, we first screened the 13 tumours with DFT1-B and DFT2-B (table 1); none of these samples amplified either marker. Next, we screened the 13 samples, together with their matched hosts, across a panel of four polymorphic microsatellites (electronic supplementary material, table S2B). None of the 13 samples showed the characteristic microsatellite profiles of DFT1 or DFT2 [2], suggesting that explanations (i) and (ii) above are unlikely to apply. Furthermore, all 12 of these samples for which matched host tissue was available showed microsatellite profiles which were identical to those of their hosts (electronic supplementary material, table S2B). Thus, it appears likely that the biopsies used for DNA extraction carried only host DNA, or that DFTD DNA was present at a low level and was undetectable under our assay conditions (explanation (iii) above). To further assess this possibility, we performed an additional DNA extraction on one of these tumours and confirmed DFT1-A positivity on this second attempt. Thus, variation in the contribution of neoplastic cells to tumour tissues should be considered in the event of an unexpected negative finding.

### 2.4. Tumour screen

We used Tasman-PCR to screen DNA from 175 suspected DFTD tumours collected from 114 devils (table 2; electronic supplementary material, table S3B). In these cases, DFTD was suspected due to gross morphology and clinical presentation, but no records of confirmed diagnosis based on histopathology, cytogenetics or microsatellite genotyping were found. Of these suspected DFTDs, 158 were positive for DFT1-A (90.3%) and five were positive for DFT2-A (2.9%) (table 2; electronic supplementary material, table S3B). None of the 12 DFT1-A and DFT2-A negative tumours were positive for DFT1-B or DFT2-B. Microsatellite genotyping confirmed that all nine DFT1-A and DFT2-A negative suspected DFTDs for which microsatellites could be amplified and for which matched host tissue was available were identical to their matched host (electronic supplementary material, table S2C). This suggests that these samples may be DFTD tumours from which only non-neoplastic host tissue was sampled for DNA extraction (explanation (iii) above); alternatively, it is possible that these samples may be non-DFTD host-derived lesions.

We next used Tasman-PCR to screen DNA from 27 confirmed or suspected non-DFTD lesions. These lesions included cutaneous lymphomas, papillomas, squamous cell carcinomas and other carcinomas (table 2; electronic supplementary material table, S3C). None of the lesions amplified either DFT1-A or DFT2-A. Microsatellite alleles were analysed in the 27 non-DFTD samples, together with their matched hosts when available. Several samples had one or two microsatellite alleles which were not detectable within the tissues of the matched host (electronic supplementary material, table S2D). However, non-DFTD lesions from different individuals had different genotypes. This suggests that the tumour-unique microsatellite alleles arose via somatic mutation of matched host alleles and does not support the possibility that any of these 27 samples represent additional transmissible cancers.

### 2.5. Distribution of DFT1 and DFT2

**Figure 2.**
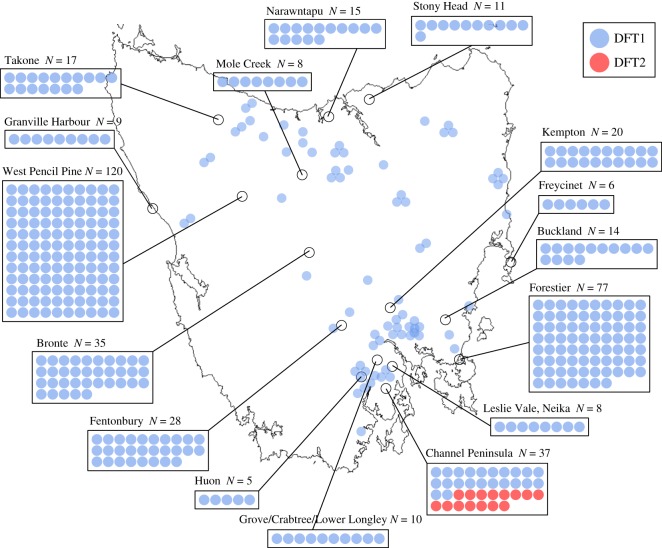
Distribution of DFT1 and DFT2. Locations of 494 tumours, sampled from 360 devils between 2003 and 2016, which amplified either DFT1-A (479 tumours) or DFT2-A (15 tumours). Each tumour is represented by a blue (DFT1) or red (DFT2) dot. The Channel Peninsula, where all DFT2-A-positive tumours were found, is located in Tasmania's southeast.

Of the 494 confirmed or suspected DFTD tumours which amplified a DFT1 or DFT2 tumour marker in Tasman-PCR, 15 tumours from 11 devils were positive for DFT2-A (electronic supplementary material, tables S3A and S3B). These DFT2-A-positive animals were all sampled in the Channel Peninsula between 2014 and 2016 (figure 2). The remaining 479 tumours, collected from 63 locations between 2003 and 2016, amplified DFT1-A (figure 2). Although we cannot rule out the possibilities that the current or historical DFT2 range extends beyond the Channel Peninsula, or that DFT2 arose prior to 2014, our results support the idea that DFT2 may have arisen recently within the Channel Peninsula.

### 2.6. DFT1 and DFT2 host gender

**Figure 3.**
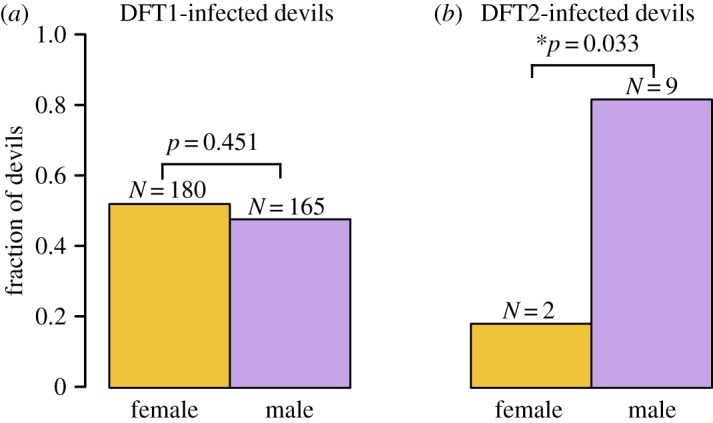
Gender distribution of DFT1 and DFT2. Gender distributions of 345 devils with one or more DFT1 tumours and 11 devils with one or more DFT2 tumours. ‘Fraction of devils’ denotes the fraction of devils hosting at least one DFT1 tumour that are male and female (left) or the fraction of devils hosting at least one DFT2 tumour that are male and female (right). The *p*-values represent outcome of Fisher's exact test for no difference in DFT1 and DFT2 host gender and * denotes significance (*p* < 0.05).

Our screen detected 11 animals with DFT2 tumours, including the five previously reported cases [2]. Of these, nine (82%), were male (Fisher's exact test for no difference in DFT2 host gender, *p* = 0.033) (figure 3). By contrast, DFT1 tumours were equally distributed between male and female hosts (of the 345 animals with one or more DFT1 tumours with known gender, 165 (47.8%) were male and 180 (52.2%) were female (Fisher's exact test for no difference in DFT1 host gender, *p* = 0.451), as previously reported [9] (figure 3). This skew towards male hosts in DFT2 suggests that this clone may preferentially affect males, although larger numbers of DFT2 cases will be required to confirm this.

### 2.7. Co-infection with DFT1 and DFT2

**Figure 4.**
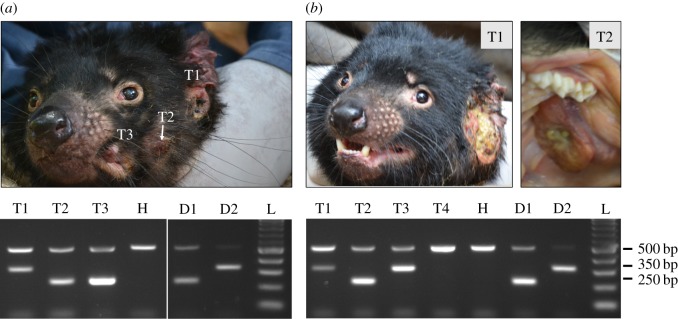
Co-infection with DFT1 and DFT2. Two devils were identified with both DFT1 and DFT2 tumours, (*a*) Devil 812 and (*b*) Devil 818. T1, T2, T3, T4 denote individual tumours from each animal, some of which are depicted in photographs. PCR results from each tumour are shown on gel and H denotes normal DNA from the matched host. D1, D2 and L denote DFT1 control, DFT2 control and ladder respectively. In Devil 818, T3 was a tumour involving the left pre-auricular lymph node and T4 was a suspected tumour found in the left submandibular lymph node.

It has previously been noted that individual devils can simultaneously harbour tumours belonging to two distinct strains, or subclones, of DFT1 [4]. As the DFT1 and DFT2 ranges overlap in the Channel Peninsula, we investigated the possibility of co-infection with DFT1 and DFT2. We identified two individuals, Devil 812 and Devil 818, which carried both DFT1 and DFT2 tumours (figure 4). Devil 812 was a 4-year-old female with three facial tumours, denoted 812T1, 812T2 and 812T3. 812T2 and 812T3 were both DFT1, whereas 812T1 was DFT2 (figure 4). Devil 818, a 2-year-old male, carried a large DFT2 tumour, 818T1, ventral to the left ear, as well as a DFT1 tumour on the hard palate, 818T2 (figure 4). Devil 818 also had a DFT2 mass in the left pre-auricular lymph node (818T3) (figure 4), probably a metastasis from 818T1.

## 3. Discussion

We present a PCR-based genetic diagnostic test for DFTD, Tasman-PCR, which complements existing diagnostic methods to provide rapid and cost-effective confirmation of DFT1 and DFT2. The test uses amplification of tumour-specific interchromosomal translocations to identify DFT1 and DFT2, and to distinguish between DFTD (DFT1 or DFT2) and non-DFTD tumours in devils. We used the assay to screen 546 devil tumours collected from more than 69 locations in Tasmania between 2003 and 2016.

Although the assay that we describe provides a useful method for diagnosis of DFT1 and DFT2, it has some limitations. Users should be aware of the potential for DNA cross-contamination to produce false positive results, and electrophoresis-based detection methods have limited sensitivity in samples with very low tumour cell fractions. DFT1 and DFT2 tumours are heterogeneous tissues which contain both neoplastic and normal cell components. While very little input material is required for PCR, which allows for minimally invasive small biopsies, it can also increase the likelihood of obtaining biopsies that contain no neoplastic cells, leading to false negative results. Moreover, although we did not find evidence of germline DFT1-A or DFT2-A in a screen of 804 normal devils, we cannot exclude the possibility that these markers are rare polymorphisms in devil populations; we suggest that users analyse matched normal tissues alongside tumours to control for this possibility. Additionally, although our screen did not identify any clear cases of DFT1 or DFT2 tumours negative for DFT1-A or DFT2-A, respectively, it is possible that such lineages may have previously arisen or may occur in future. Users encountering unexpected negative results should perform PCR for additional markers, such as DFT1-B and DFT2-B (table 1) as well as repeating DNA extraction and PCR from a separate biopsy of the same tumour. Importantly, this assay is designed to be complementary with other established DFT1 and DFT2 diagnostic methods, such as histology and cytogenetics, while being rapid, scalable and minimally invasive. Furthermore, it would be possible to develop this method to obtain a real-time diagnosis in the field with the use of portable PCR laboratories [17,18].

The discovery of two transmissible cancers in Tasmanian devils raises the possibility that additional, so far undetected, transmissible cancer clones exist within devil populations. In this study, we screened 546 Tasmanian devil tumours, collected between 2003 and 2016 from more than 69 locations in Tasmania (table 3). In total, 479 (87.7%) were DFT1 and 15 (2.7%) were DFT2; the remaining 52 (9.5%) were either confirmed or suspected host-derived non-DFTD lesions (27, 4.9%) or biopsies that shared identical microsatellite profiles with their matched hosts (as well as four biopsies for which matched host tissue was unavailable or microsatellites could not be amplified) (25, 4.5%) (table 3). Thus, this screen has not detected evidence for additional transmissible cancers in Tasmanian devils. However, the method provides a useful tool for devil tumour monitoring and surveillance.

**Table 3.** Results of screen of 546 Tasmanian devil tumours. ‘No information on similarity to matched host’ denotes tumours for which matched host DNA was unavailable or for which microsatellite loci failed to amplify.

marker amplified	number of tumours
DFT1-A	479
DFT2-A	15
*RPL13A* only	52
	27 non-DFTD lesions 21 microsatellite alleles identical to matched host 4 no information on similarity to matched host
total	546

DFT1 had been detected in the Channel Peninsula prior to the discovery there of DFT2, and previous studies have shown that different DFT1 strains, or subclones, can co-infect one host [4]. Here, we confirmed that DFT1 and DFT2 can also co-infect the same devil. Co-infections of these two phenotypically similar DFTs highlight the importance of careful monitoring, as competition for a limited host devil population may influence the evolution of virulence [19].

Of the 11 DFT2 cases that we detected, nine involved a male host (Fisher's exact test for no difference in DFT2 host gender, *p* = 0.033). This apparent preference for male hosts may reflect underlying transmission dynamics. Alternatively, it is possible that females are less susceptible to DFT2, perhaps due to the recognition of Y chromosome-derived allogeneic antigens [8]. Nevertheless, we detected two females with DFT2 tumours, confirming that this disease does not exclusively affect males. The gender bias observed in DFT2 may impact devil population structures by reducing overall male devil abundance; over time, this may decrease reproductive success and increase inbreeding by limiting mating opportunities. Furthermore, if DFT2 is largely excluded from female hosts, this may affect the future potential for this clone to survive, perhaps selecting for the emergence of DFT2 subclones with balanced host gender proclivity through Y chromosome loss [8].

Overall, this study presents Tasman-PCR, a PCR-based assay for rapid, high-throughput diagnosis of DFT1 and DFT2 in Tasmanian devils. By screening 546 tumours, we have documented the distributions of DFT1 and DFT2, and identify two devils co-infected with both clones. Our analysis does not provide evidence for additional DFTD clones beyond DFT1 and DFT2. The co-occurrence of two transmissible cancer clones in Tasmanian devils presents unique challenges for the conservation of this iconic species. The genetic diagnostic screening tools described here provide important additional resources for population monitoring and disease surveillance.

## 4. Material and methods

### 4.1. Sample collection and DNA extraction

Biopsies were collected from wild or captive Tasmanian devils or from animals whose carcasses were found as roadkill. Tumour and host (ear, liver, spleen, blood, gonad or submandibular lymph node) tissue biopsies were collected into either RNAlater or ethanol. Fine needle aspirates collected into a 1 : 1 solution of Buffer AL (Qiagen) : PBS were sampled from tumours that were too small to biopsy. A subset of tumours were previously confirmed as DFT1 or DFT2 using histopathology, cytogenetics or microsatellite genotyping [9,10,16]. Genomic DNA was extracted using the Qiagen DNeasy Blood and Tissue Kit. Genomic DNA was amplified using the Illustra GenomiPhi V2 DNA Amplification Kit (GE Healthcare) prior to PCR amplification.

### 4.2. Selection of markers

Two markers for DFT1 (DFT1-A and DFT1-B) and two markers for DFT2 (DFT2-A and DFT2-B) were evaluated (table 1). All four markers amplified products that were DFT1/DFT2-specific in a preliminary screen. DFT1-A and DFT2-A were selected for development of the triplex assay; DFT1-B and DFT2-B can be used to provide additional diagnostic confirmation if required.

### 4.3. Tasman-PCR amplification and gel electrophoresis

Primers used in this study are as follows:

**Table:** 

marker	location	expected product length	sequence (5′ to 3′)
DFT1-A	Chr2_GL841420:ChrX_GL867598	231 bp	AGTAAAAATGCAATAGGCCCAGG
			GGTGATGGCAGATTTCAGCTAAG
DFT1-B	Chr1_GL834488:Chr4_GL856767	284 bp	GTACTGATTTCCTGCCAGTCTCTT
			GGAACAATAGCATTGGTAAAGGG
DFT2-A	Chr4_GL856969:Chr5_GL861630	321 bp	AGGGGATCATTCATAGAGAACACTT
			CTATAGCTGATCTATGGGAAGACAATG
DFT2-B	Chr4_GL856736:Chr5_GL861700	360 bp	GAAGGATATTGTATCTTCAGATGGC
			ATAATCTCTTCTCGCTTAAGGTGACTC
normal (*RPL13A* locus)	Chr3_GL849778	520 bp	CCCCACAAGACCAAGCGAGGC
			ACAGCCTGGTATTTCCAGCCAACC

The triplex PCR was performed in 20 µl reactions with 2 units Qiagen Taq Polymerase, 1X CoralLoad PCR buffer, 0.2 mM each dNTP and approximately 20 ng genomic DNA per reaction. Primers (Sigma-Aldrich) were at a final concentration of 0.4 µM (DFT1-A and DFT2-A primers) or 0.8 µM (*RPL13A* primers). Amplification was performed using a Nexus GS-1 Thermal Cycler (Eppendorf) with the following conditions:

**Table:** 

temperature (°C)	time	cycles
94	3 min 30 s	1
94	30 s	30
60	30 s	
72	30 s	
72	5 min	1

Single (non-triplex) PCRs, including those involving DFT1-B and DFT2-B, were performed with the same conditions, with primers at a final concentration 0.4 µM. Gel electrophoresis was performed with a 3% agarose gel and products were detected using ethidium bromide. Samples with weak amplification bands were identified, and PCR was repeated with increased DNA input.

### 4.4. Microsatellite genotyping

Microsatellite amplification and fragment size detection was performed across four loci (C, D, N and F) as previously described [2] and analysed using Fragman [20].

## Supplementary Material

Table S1

## Supplementary Material

Table S2

## Supplementary Material

Table S3

## Data accessibility

Data are available in electronic supplementary material, tables S1–S3.

## References

[1] Pearse AM, Swift K 2006 Allograft theory: transmission of devil facial-tumour disease. Nature 439, 549 (10.1038/439549a) 16452970

[2] Pye RJ et al. 2016 A second transmissible cancer in Tasmanian devils. Proc. Natl Acad. Sci. USA 113, 374–379. (10.1073/pnas.1519691113) 26711993 PMC4720317

[3] Deakin JE et al. 2012 Genomic restructuring in the Tasmanian devil facial tumour: chromosome painting and gene mapping provide clues to evolution of a transmissible tumour. PLoS Genet. 8, e1002483 (10.1371/journal.pgen.1002483) 22359511 PMC3280961

[4] Murchison EP et al. 2012 Genome sequencing and analysis of the Tasmanian devil and its transmissible cancer. Cell 148, 780–791. (10.1016/j.cell.2011.11.065) 22341448 PMC3281993

[5] Hawkins CE et al. 2006 Emerging disease and population decline of an island endemic, the Tasmanian devil *Sarcophilus harrisii*. Biol. Conserv. 131, 307–324. (10.1016/j.biocon.2006.04.010)

[6] Lazenby BT et al. 2018 Density trends and demographic signals uncover the long-term impact of transmissible cancer in Tasmanian devils. J. Appl. Ecol. 55, 1368–1379. 30089931 10.1111/1365-2664.13088 PMC6078421

[7] McCallum H 2008 Tasmanian devil facial tumour disease: lessons for conservation biology. Trends Ecol. Evol. 23, 631–637. (10.1016/j.tree.2008.07.001) 18715674

[8] Stammnitz MR et al. 2018 The origins and vulnerabilities of two transmissible cancers in Tasmanian devils. Cancer Cell. 33, 607–619. (10.1016/j.ccell.2018.03.013) 29634948 PMC5896245

[9] Loh R, Bergfeld J, Hayes D, O'Hara A, Pyecroft S, Raidal S, Sharpe R 2006 The pathology of devil facial tumor disease (DFTD) in Tasmanian Devils (*Sarcophilus harrisii*). Vet. Pathol. 43, 890–895. (10.1354/vp.43-6-890) 17099145

[10] Murchison EP et al. 2010 The Tasmanian devil transcriptome reveals Schwann cell origins of a clonally transmissible cancer. Science 327, 84–87. (10.1126/science.1180616) 20044575 PMC2982769

[11] Tovar C, Obendorf D, Murchison EP, Papenfuss AT, Kreiss A, Woods GM 2011 Tumor-specific diagnostic marker for transmissible facial tumors of Tasmanian devils: immunohistochemistry studies. Vet. Pathol. 48, 1195–1203. (10.1177/0300985811400447) 21383118

[12] Siddle HV et al. 2013 Reversible epigenetic down-regulation of MHC molecules by devil facial tumour disease illustrates immune escape by a contagious cancer. Proc. Natl Acad. Sci. USA 110, 5103–5108. (10.1073/pnas.1219920110) 23479617 PMC3612627

[13] Metzger MJ, Reinisch C, Sherry J, Goff SP 2015 Horizontal transmission of clonal cancer cells causes leukemia in soft-shell clams. Cell 161, 255–263. (10.1016/j.cell.2015.02.042) 25860608 PMC4393529

[14] Metzger MJ, Villalba A, Carballal MJ, Iglesias D, Sherry J, Reinisch C, Muttray AF, Baldwin SA, Goff SP 2016 Widespread transmission of independent cancer lineages within multiple bivalve species. Nature 534, 705–709. (10.1038/nature18599) 27338791 PMC4939143

[15] Murchison EP 2008 Clonally transmissible cancers in dogs and Tasmanian devils. Oncogene 27(Suppl. 2), S19–S30. (10.1038/onc.2009.350) 19956175

[16] Pearse AM, Swift K, Hodson P, Hua B, McCallum H, Pyecroft S, Taylor R, Eldridge MDB, Belov K 2012 Evolution in a transmissible cancer: a study of the chromosomal changes in devil facial tumor (DFT) as it spreads through the wild Tasmanian devil population. Cancer Genet. 205, 101–112. (10.1016/j.cancergen.2011.12.001) 22469509

[17] Koo C et al. 2013 Development of a real-time microchip PCR system for portable plant disease diagnosis. PLoS ONE 8, e82704 (10.1371/journal.pone.0082704) 24349341 PMC3861469

[18] Taylor BJ et al. 2014 A lab-on-chip for malaria diagnosis and surveillance. Malar. J. 13, 179 (10.1186/1475-2875-13-179) 24885206 PMC4029813

[19] Alizon S, Hurford A, Mideo N, Van Baalen M 2009 Virulence evolution and the trade-off hypothesis: history, current state of affairs and the future. J. Evol. Biol. 22, 245–259. (10.1111/j.1420-9101.2008.01658.x) 19196383

[20] Covarrubias-Pazaran G, Diaz-Garcia L, Schlautman B, Salazar W, Zalapa J 2016 Fragman: an R package for fragment analysis. BMC Genet. 17, 62 (10.1186/s12863-016-0365-6) 27098093 PMC4839125

